# Design and Validation of an Impaction-Nozzle Nebuliser for Enhanced Distribution Uniformity in Pressurised Intraperitoneal Aerosol Chemotherapy (PIPAC) Applications

**DOI:** 10.1245/s10434-025-17602-4

**Published:** 2025-06-25

**Authors:** Yaroslaw Sautkin, Hans Schoenfelder, Marc André Reymond

**Affiliations:** 1 National Center for Pleura and Peritoneum, University of Tuebingen, Tuebingen, Germany; 2 CapnoPharm GmbH, Tuebingen, Germany

**Keywords:** PIPAC, Doxorubicin, Cisplatin, CapnoTip^®^, CapnoPen^®^, Aerosol chemotherapy, Peritoneal carcinomatosis, Peritoneal metastasis

## Abstract

**Introduction:**

This study introduces and validates the CapnoTip^®^, an impaction-based pressurised intraperitoneal aerosol chemotherapy (PIPAC) nebuliser designed to enhance intraperitoneal drug delivery and achieve greater homogeneity to improve treatment outcomes for peritoneal metastasis.

**Methods:**

CapnoTip^®^ was characterised through physical experiments evaluating aerosol granulometry, spray patterns, and aerosolisation angle. Pharmacological efficacy was assessed by using the ex vivo enhanced inverted bovine urinary bladder (eIBUB) model to measure intraperitoneal cisplatin concentration and distribution homogeneity and compare the result to that of the clinical reference nebuliser, CapnoPen^®^.

**Results:**

Aerosol granulometry using distilled water measured 26.1 µm (confidence interval [CI] 13.6–59.6) for CapnoTip^®^ and 27.9 µm (CI 14.8–59.4) for CapnoPen^®^. When using 10 cSt silicone oil, droplet sizes were 56.0 µm (CI 18.4–245.0) for CapnoTip^®^ versus 33.8 µm (CI 14.3–66.5) for CapnoPen^®^. The aerosolisation angle was broader with the CapnoTip^®^ compared with the CapnoPen^®^ (155.3° vs. 67.1°). CapnoTip^®^ achieved a uniform intraperitoneal drug distribution, with no significant cisplatin gradient along the aerosolisation axis (*p* > 0.05). In contrast, CapnoPen^®^ showed marked concentration gradients between the test organ’s top vs. bottom and middle vs. bottom regions (*p* < 0.001). A significantly higher mean intraperitoneal cisplatin concentration was achieved with the CapnoTip^®^ (56.8 ± 25.1 ng/mg) compared with the CapnoPen^®^ (39.2 ± 31.1 ng/mg; *p* = 0.026).

**Conclusions:**

The CapnoTip^®^ impaction-nozzle nebuliser for PIPAC is technically and pharmacologically equivalent to the CE-approved CapnoPen^®^, while offering superior intraperitoneal drug delivery and distribution homogeneity.

Pressurised intraperitoneal aerosol chemotherapy (PIPAC) represents an innovative, minimally invasive approach for treating peritoneal metastasis, offering enhanced drug delivery and more uniform distribution across the affected peritoneal surface compared with liquid chemotherapy.^1–3^ Pressurised intraperitoneal aerosol chemotherapy delivers a therapeutic aerosol, generated via a single-use, sterile laparoscopic nebuliser, directly into the CO₂-expanded abdominal cavity.^4^ Compared with traditional liquid-based chemotherapy, PIPAC has shown superior pharmacokinetics, achieving deeper tissue drug penetration and higher local drug concentrations.^5,6^ Despite these advantages, drug distribution with PIPAC remains uneven, with notable gradients across the intra-abdominal cavity.^6,7^

Aerosol generation within PIPAC is influenced by various factors, including injection flow rate, aerosolisation angle, and pressure, as well as the properties of the chemotherapeutic agents.^8–10^ Gravity also plays a significant role in the aerosol’s behaviour, promoting sedimentation and liquid pooling, which can hinder homogeneous drug delivery.^11^ Uneven drug distribution can limit exposure of some tumour regions, potentially impacting the overall efficacy of the treatment.^3^

Advancements in PIPAC technology are required to improve the uniformity of drug delivery.^8,12,13^ Our team previously developed a multinozzle nebuliser, adding two side nozzles to the standard CapnoPen^®^ nebuliser to enhance tissue drug exposure and delivery. However, preclinical data showed that the three-nozzle configuration decreased tissue drug concentration and distribution uniformity compared with the single-nozzle CapnoPen^®^. This was likely due to increased injection flow and reduced aerosolisation time, which caused rapid aerosol oversaturation, promoting larger particle formation, sedimentation, and liquid pooling.^9^

Given these challenges, we sought to refine the CapnoPen^®^ by developing an impaction-based, single-nozzle nebuliser, CapnoTip^®^, which maintains optimal droplet size, flow rate, and aerosolisation time. This modification aims to enhance intraperitoneal drug delivery by achieving a more stable and evenly distributed aerosol. The primary objective of this study is to validate CapnoTip^®^’s efficacy in delivering drugs more homogeneously across the peritoneal surface than the current clinical standard, CapnoPen^®^. Through rigorous in vitro and ex vivo evaluations, we strive to establish CapnoTip^®^ as an optimised solution for more efficient intraperitoneal drug delivery.

## Methods

In this study, we developed and preclinically validated CapnoTip^®^, an impaction-based PIPAC nebuliser, to evaluate its potential for improving intraperitoneal drug delivery and distribution uniformity compared with the clinical standard, CapnoPen^®^. We hypothesised that CapnoTip^®^ would provide greater and more uniform drug delivery than CapnoPen^®^.

### Study Design

Physical and pharmacological assessments were conducted to compare the performance of CapnoTip^®^ and CapnoPen^®^. Physical testing was performed on a technical platform to evaluate aerosolisation parameters, aerosol particle size (granulometry), spray distribution, and aerosolisation angle. Pharmacological evaluation employed the ex vivo enhanced Inverted Bovine Urinary Bladder Model (eIBUB) to quantify real-time drug delivery, tissue drug concentrations, and distribution homogeneity within the peritoneum.

### Sample Size

We determined the sample size to exclude a 30% difference in cisplatin concentration, which would be considered clinically significant. For this purpose, we used pilot data after PIPAC with cisplatin, with a mean cisplatin concentration of 23.4 ng/mg tissue, and a standard deviation of 9.2.^9^ The effect size to exclude was 30% of 23.4 = 7.02. The calculated effect size (Cohen’s d) was ≈ 0.763. Assuming Type I error *α* = 0.05, a power (1−*β*) = 0.8, and choosing a two-sample *t*-test using the standard sample size formula for a two-sample *t*-test, the approximate sample size needed was 27 per group. Since nine biopsies are taken from a single test organ, three organs per group were needed.

### Occupational Health and Safety

All experiments were conducted at the CapnoPharm R&D facility, an S1 laboratory certified by the German Workers’ Health Insurance (BG-RCI) for the safe handling of cytotoxic aerosols. The facility is equipped with a Class II cytotoxic-approved safety cabinet, providing a controlled environment with continuous laminar airflow. Regular annual inspections are performed to monitor surface and airborne contamination. All staff adhere to strict safety protocols, are certified in Good Laboratory Practice (GLP) and Good Scientific Practice (GSP) and undergo annual occupational health examinations.

### Ethical and Regulatory Approvals

This study did not involve any in vivo animal experiments. The ex vivo experiments were authorised by the local Veterinary Office (registration number: DE08416120321) and conducted using animal tissue obtained from routine slaughter, thereby exempting the study from additional ethical approval requirements. No human biological materials or personal data were used.

### PIPAC Nebulisers

*CapnoPen*^*®*^ The CapnoPen^®^ CP-001 (CapnoPharm GmbH, Tuebingen, Germany) is a single-nozzle PIPAC nebuliser with a high-strength polyvinyl chloride (PVC) line capable of sustaining a pressure of 45 bar. It is certified in the EC as a Medical Device Regulation (MDR) Class IIb device for various aerosol therapies, including chemotherapy, viral applications, gene therapy, nanoparticles, and antibodies for immunotherapy.

*CapnoTip*^*®*^ CapnoTip^®^ (CapnoPharm GmbH, Tuebingen, Germany) is a modified version of CapnoPen^®^, implementing impaction technology in a single-nozzle configuration. It features a wider aerosolisation angle to enhance drug delivery and distribution uniformity by reducing aerosol oversaturation.

### PIPAC Drug Agents

Doxorubicin (DOX) and cisplatin (CIS), standard agents in PIPAC, were used for this study. Doxorubicin (2.7 mg) was diluted in 50 mL of 0.9% saline, while CIS (13.5 mg) was prepared in 150 mL of 0.9% saline. Both solutions were stored in 200-mL syringes sealed with Luer-lock stoppers until used in PIPAC.

### PIPAC Procedure

The PIPAC system comprised the nebuliser, a high-pressure injector, and the drug solutions. The Accutron HP-D (Medtron AG, Saarbruecken, Germany) was used in our setup to pressurise the drug solutions, driving them through CapnoPen^®^ or CapnoTip^®^ for aerosolisation.

### Physical Experiments

The performance of CapnoPen^®^ and CapnoTip^®^ was compared across varying parameters to optimise aerosolisation angle, surface spray coverage, and aerosol particle size and homogeneity. Conditions included injection pressures of 10–20 bars, flow rates of 0.2–1.3 mL/s, and solution viscosities of 1–10 cSt. Aerosolisation time and angle were documented for each device.

*Granulometry* Aerosol particle size and size distribution were measured using laser diffraction spectroscopy (Spraytec™, Malvern Panalytical GmbH, Kassel, Germany). Each nebuliser was positioned above the laser, and 50 mL of distilled water, 5% glucose, and 5 and 10 cSt silicon oil were aerosolised in triplicate.

**Fig. 1:**
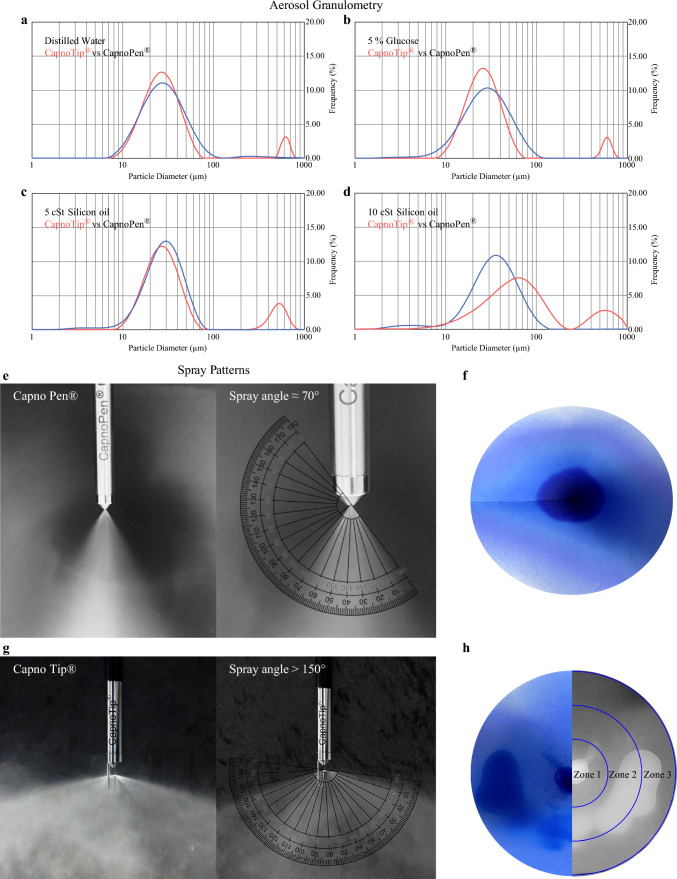
Results of physical experiments. Aerosol granulometry: (a–d). (**a**) Median aerodynamic diameter (MAD) of aerosol generated with distilled water using CapnoTip^®^ (red line) and CapnoPen^®^ (black line). (**b**) MAD of aerosol generated with 5% glucose using CapnoTip^®^ (red line) and CapnoPen^®^ (black line). (**c**) MAD of aerosol generated with 5 cSt silicon oil using CapnoTip^®^ (red line) and CapnoPen^®^ (black line). (**d**) MAD of aerosol generated with 10 cSt silicon oil using CapnoTip^®^ (red line) and CapnoPen^®^ (black line). *Results indicate that aerosol particle sizes were comparable between CapnoTip*^*®*^* and CapnoPen*^*®*^* when nebulising distilled water and 5% glucose. However, CapnoTip*^*®*^* generated larger particles when nebulising 5 cSt and 10 cSt silicone oils, reflecting differences in fluid dynamics with higher-viscosity substances*. Spray Patterns: e-h. **e** Angle of aerosolisation with CapnoPen^®^ nebulising distilled water. (**f**) Surface ink coverage of the conic folded blotting paper after aerosolisation with CapnoPen^®^. (**g**) Angle of aerosolisation with CapnoTip^®^ nebulising distilled water. (**h**) Surface ink coverage of the conic folded blotting paper after aerosolisation with CapnoTip^®^, with the right half showing an example of three measurement zones (centre to periphery) for relative integrated staining density. *CapnoTip*^*®*^* exhibited an aerosolisation angle more than twice that of CapnoPen*^*®*^*, resulting in broader and more intensive surface coverage. This was evidenced by blue ink distribution patterns, with CapnoTip*^*®*^* leaving significantly fewer unimpacted white areas, particularly at the periphery.*

*Spatial spray patterns* Distribution patterns were visualised by aerosolising 50 mL of blue ink onto conically folded blotting paper (Whatman^®^ GB005, Cytiva, Marlborough, MA). Nebulisers were positioned at the cone base, and images were captured immediately after aerosolisation and analysed using ImageJ™ software (National Institutes of Health, Bethesda, MD) to determine the relative integrated staining density across three zones (Fig. 1h).

*Angle of aerosolisation* The aerosolisation angle was photo-documented from a distance of 30 cm from the nozzle tip using a 12-megapixel camera, while nebulising 100 mL of distilled water at optimal pressure. Following 8-bit greyscale conversion, the angle was digitally measured using a protractor tool (Fig. 1e, g).

### Pharmacological Experiments

The intraperitoneal drug delivery and distribution characteristics of CapnoTip^®^ were validated preclinically using the eIBUB model, an established framework for pharmacological PIPAC studies and device optimisation for peritoneal application. The eIBUB model leverages the bovine urinary bladder’s anatomical and histological similarities to the human peritoneum, including a volume of 3–4 litres, comparable to that of the human abdominal cavity, making it suitable for testing intraperitoneal drug delivery dynamics.

### eIBUB Model Setup

Bovine urinary bladders were obtained from a local abattoir and transported to the laboratory immediately postexplantation. Each bladder was rinsed with water, cleared of extraneous tissue, weighed, and inverted. The bladder base was sealed to an airtight plastic cup using the principle of communicating vessels to collect aerosol sediment. A 15-mm trocar was inserted at the bladder neck to allow intraluminal access for the nebuliser. The eIBUB model was placed on precision scales to monitor weight changes during aerosolisation, with increasing weight reflecting real-time tissue drug absorption. The plastic cup collected nondeposited aerosol sediment, providing a quantifiable measure of nontherapeutic drug loss. For each nebuliser, three bladders were used per experimental run.

### Real-Time Intraperitoneal Drug Delivery

Weight increase in the eIBUB model was recorded in real-time as an indicator of liquid drug uptake into the tissue. The difference between the applied and sedimented drug volume further quantified tissue drug delivery, with calculated values provided for each experiment.

### Intraperitoneal Drug Concentrations

Following PIPAC, three biopsies were taken from each bovine urinary bladder's top, middle, and bottom regions to measure CIS concentrations. The samples were stored at −80 °C until analysis. A total of 27 biopsies were collected for each nebuliser to validate pharmacological outcomes.

### Homogeneity of Drug Distribution

Drug distribution uniformity was assessed by measuring the vertical gradient of CIS concentrations across each bovine urinary bladder’s top, middle, and bottom biopsies, comparing the distribution profiles between CapnoPen^®^ and CapnoTip^®^.

### Preanalytical Biopsy Preparation

Biopsies from the eIBUB model were freeze-dried for 24 hours at room temperature under a vacuum of 3.0 mbar using an Alpha 1-2 LDplus lyophiliser (Martin Christ Gefriertrocknungsanlagen GmbH, Osterode am Harz, Germany). Dried samples were weighed, transferred into 2-mL PowerBead™ tubes (QIAGEN GmbH, Hilden, Germany) and rehydrated with 1.5 mL of distilled water for 24 h at 4 °C. Samples were then homogenised with a TissueLyser LT (QIAGEN GmbH, Hilden, Germany) at 50 Hz for 5 h, centrifuged at 11,000 rpm, and stored at −80 °C until analysis.

### Analysis of Cisplatin Concentrations in Tissue

Cisplatin concentrations in eIBUB tissue biopsies were determined using atomic absorption spectroscopy (AAS; ZEEnit P 650, Analytic Jena AG, Jena, Germany) at a GLP-certified facility (Medizinisches Versorgungszentrum Dr. Eberhard & Partner, Dortmund, Germany). Preanalytical validation confirmed a lower quantification limit of 50 ng/mL with no interference from organic matrices.

### Statistical Analysis

All experiments were conducted in accordance with internal standard operating procedures (SOPs), and all data were securely stored on the company’s server. Statistical analyses were performed using SPSS™ (IBM, NY, USA). Descriptive statistics were presented as mean, median, standard deviation, and 95% confidence intervals. Because of nonnormal data distribution, comparative analyses were performed by using one-way ANOVA and post hoc testing. *P* < 0.05 was considered statistically significant.

## Results

**Fig. 2:**
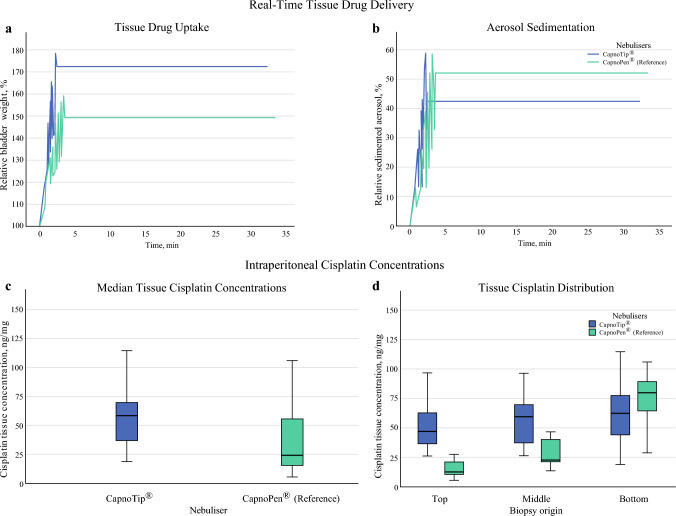
Results of Pharmacological Experiments. Real-time Tissue Drug Delivery: (a, b). (**a**) Real-time tissue drug uptake during PIPAC with CapnoTip^®^ (blue line) and CapnoPen^®^ (green line). *CapnoTip*^*®*^* showed significantly greater intraperitoneal drug delivery than CapnoPen*^*®*^* (one-way ANOVA, p < 0.001)*. (**b**) Real-time aerosol sedimentation during PIPAC with CapnoTip^®^ (blue line) and CapnoPen^®^ (green line). *Aerosol sedimentation was lower with CapnoTip*^*®*^* than with CapnoPen*^*®*^*, although this result was not statistically significant (one-way ANOVA, p = 0.051)*. Intraperitoneal Cisplatin Concentrations: (c, d). (**c**) Median intraperitoneal cisplatin concentrations after PIPAC with CapnoTip^®^ (blue column) and CapnoPen^®^ (green column). *The mean cisplatin concentration in the peritoneal tissue was significantly higher with CapnoTip*^*®*^* than with CapnoPen*^*®*^* (one-way ANOVA, p = 0.026).* (**d**) Intraperitoneal cisplatin distribution along the aerosolisation axis (top, middle, bottom) of the ex vivo peritoneal cavity after PIPAC with CapnoTip^®^ (blue column) and CapnoPen^®^ (green column). *CapnoTip*^*®*^* demonstrated a more homogeneous cisplatin distribution in the peritoneal tissue with no vertical gradient along the nebulisation axis (post hoc, p > 0.05), contrasting with the significant gradient observed with CapnoPen*^*®*^* (post hoc, top vs. bottom and middle vs. bottom p < 0.001)*

The CapnoTip^®^ nebuliser was technically characterised and pharmacologically validated compared with the clinical reference device, CapnoPen^®^ (Figs. 1 and 2).

### Physical Experiments

The technical characterisation of CapnoTip^®^ and CapnoPen^®^ included measurements of aerosol granulometry, spatial spray patterns, and the angle of aerosolisation.

## Granulometry

Median aerosol particle sizes for various solutions were as follows: for distilled water, CapnoTip^®^ produced particles of 26.1 µm (CI 13.6–59.6), whereas CapnoPen^®^ produced 27.9 µm (CI 14.8–59.4) (Fig. 1a); for 5% glucose, CapnoTip^®^ yielded 26.6 µm (CI 13.6–69.7) compared with CapnoPen^®^’s 28.2 µm (CI 13.2–61.6) (Fig. 1b); for 5 cSt silicon oil, CapnoTip^®^ generated 35.5 µm (CI 18.4–234.6) whereas CapnoPen^®^ generated 30 µm (CI 16.4–51.5) (Fig. 1c); and for 10 cSt silicon oil, CapnoTip^®^ produced 56 µm (CI 18.4–245) compared with CapnoPen^®^’s 33.8 µm (CI 14.3–66.5) (Fig. 1d). Parameters of injection flow (1–1.2 mL/s for CapnoTip^®^; 0.9–1 mL/s for CapnoPen^®^) and pressure (17–20 bar for CapnoTip^®^; 20 bar for CapnoPen^®^) were maintained within recommended ranges. CapnoTip^®^ and CapnoPen^®^ showed comparable particle sizes for water-based solutions, although CapnoTip^®^ produced larger particles with higher viscosity silicon oils.

### Spatial Spray Patterns

Spray distribution analysis showed that CapnoTip^®^ achieved a greater periphery exposure than CapnoPen^®^. The relative distribution of stained pixels from the centre to the periphery on a cone-shaped blotting paper was as follows: CapnoTip^®^—14.9% (CI 8.9–20.9) in zone 1, 39.8% (CI 21.6–58) in zone 2, and 45.3% (CI 21.2–69.5) in zone 3; CapnoPen^®^—18.3% (CI 11.8–24.8) in zone 1, 41.4% (CI 28–54.7) in zone 2, and 40.4% (CI 32.4–48.3) in zone 3 (Figs. 1f and h). There was no statistically significant difference between the devices across all zones (one-way ANOVA, *p* > 0.05), indicating comparable spatial spray patterns, with CapnoTip^®^ showing more extensive peripheral coverage.

### Angle of Aerosolisation

CapnoTip^®^ displayed a considerably (2.3 times) larger aerosolisation angle (155.3° for distilled water) compared with CapnoPen^®^ (67.1°) (Fig. 1e and g). When the solution viscosity was increased to 10 cSt, the angle narrowed to 131.7° for CapnoTip^®^ and 45.3° for CapnoPen^®^, representing reductions of 15% and 33%, respectively. These results suggest that CapnoTip^®^ offers broader spray patterns even for high-viscosity solutions, which is particularly beneficial for advanced formulations such as lipid emulsions or pH-sensitive gels.

Overall, the physical experiments demonstrated that CapnoTip^®^ and CapnoPen^®^ produced aerosol with comparable particle sizes for water-based solutions, but CapnoTip^®^ achieved a wider aerosolisation angle and more extensive surface spray coverage, particularly with viscous solutions.

### Biological Equivalence

The CapnoTip^®^ nebuliser demonstrates biological equivalence to the CapnoPen^®^ in accordance with MDR 2017/745, Annex XIV, Part A. Because both devices are made from identical materials, with no new substances introduced to the patient or drug solution, and are used under equivalent clinical conditions, no additional biocompatibility testing is required.

### Pharmacological Experiments and Clinical Equivalence

Pressurised intraperitoneal aerosol chemotherapy with DOX and CIS was conducted by using the ex vivo eIBUB model to evaluate tissue drug uptake, concentration, and distribution homogeneity between CapnoTip^®^ and CapnoPen^®^ (Fig. 2).

### Real-Time Intraperitoneal Drug Delivery

Real-time tissue drug uptake was significantly higher with CapnoTip^®^ (172.5 ± 19.1%) compared with CapnoPen^®^ (149.4 ± 7.7%) (one-way ANOVA, *p* < 0.001) (Fig. 2a). Aerosol sedimentation was slightly lower with CapnoTip^®^ (42.4 ± 19.1%) than with CapnoPen^®^ (52.1 ± 10.8%) (*p* = 0.051) (Fig. 2b). Both devices delivered approximately 100 mL of drug agents intraperitoneally during the aerosolisation phase, with CapnoTip^®^ demonstrating reduced aerosol sedimentation and improved tissue drug uptake.

### Intraperitoneal Drug Concentrations

Mean CIS concentrations in tissue were 56.8 ± 25.1 ng/mg with CapnoTip^®^ and 39.2 ± 31.1 ng/mg with CapnoPen^®^ (one-way ANOVA, *p* = 0.026), indicating 45% higher tissue drug concentrations with CapnoTip^®^ (Fig. 2c). This enhancement suggests that CapnoTip^®^ facilitates more efficient drug delivery to the target tissue.

### Homogeneity of Drug Distribution

Cisplatin concentration gradients across the top, middle, and bottom eIBUB biopsies were largely eliminated with CapnoTip^®^ compared with CapnoPen^®^. With CapnoTip^®^, CIS concentrations were 51.5 ± 23.5 ng/mg (top), 57.3 ± 22.5 ng/mg (middle), and 61.7 ± 30.7 ng/mg (bottom), with no significant difference among the regions (post hoc, *p* > 0.05). In contrast, CapnoPen^®^ exhibited pronounced gradients: 15.1 ± 7.2 ng/mg (top), 28.1 ± 12.3 ng/mg (middle), and 74.5 ± 27.2 ng/mg (bottom) (post hoc, top vs. bottom and middle vs. bottom, *p* < 0.001) (Fig. 2d). These results demonstrate that CapnoTip^®^ enables a more uniform intraperitoneal drug spread than CapnoPen^®^. The characteristic gradient observed with CapnoPen^®^—with minimal concentrations in the upper regions and maximal concentrations in the lower regions of the test organ—was no longer present when using CapnoTip^®^. Overall, pharmacological experiments proved that CapnoTip^®^ achieves higher and more homogeneous intraperitoneal drug delivery than CapnoPen^®^.

## Discussion

In this study, we successfully engineered and validated the CapnoTip^®^ PIPAC nebuliser, demonstrating its superiority over the clinical standard, CapnoPen^®^, in enhancing intraperitoneal drug delivery and distribution homogeneity. The physical experiments highlighted that while CapnoTip^®^ and CapnoPen^®^ share similar aerosolisation parameters, CapnoTip^®^ allows a much wider aerosolisation angle (exceeding 150°), effectively expanding the spatial spray distribution without increasing injection flow or reducing nebulisation time. This improvement addresses a limitation observed with previous multinozzle design, where aerosol oversaturation led to greater sedimentation, liquid formation, and decreased tissue drug delivery.^9^

Both CapnoTip^®^ and CapnoPen^®^ successfully aerosolised high-viscosity solutions (up to 10 cSt), demonstrating potential utility for lipid-based solutions, such as emulsions. Whereas CapnoPen^®^ produced slightly smaller aerosol particles, CapnoTip^®^ showed a much wider spray angle. This suggests that CapnoTip^®^ may be especially beneficial in applications where broad surface drug coverage is essential while maintaining the capability to handle a range of solution viscosities.

The pharmacological data demonstrated the superiority of CapnoTip^®^, achieving significantly higher mean cisplatin concentrations in peritoneal tissue compared to CapnoPen^®^. The observed enhanced local drug delivery in the upper and lateral anatomical areas of the target organ is coherent with the wider aerosolisation angle and suggests a promising strategy for dose intensification, particularly in difficult-to-reach regions. By facilitating higher local drug concentrations, CapnoTip^®^ may improve therapeutic efficacy by reducing recurrence rates in anatomically challenging areas, including the upper and lateral abdominal walls. However, only future clinical studies can determine the potential positive impact of CapnoTip^®^ on tumour regression.

Alternatively, CapnoTip^®^’s ability to deliver higher intraperitoneal drug concentrations might open the possibility of dose reduction in chemotherapy, because a smaller amount of the drug may be sufficient to reach a cytotoxic level at remote tumour sites. Reducing the chemotherapy dose could further decrease local and systemic side effects, improving the treatment’s tolerability.^14,15^ Treatment tolerability is essential in patients with peritoneal metastasis, who are often in a reduced general condition and have a limited ability to tolerate continued chemotherapy.^16^ The potential of such dose reduction should be tested in adequately powered clinical trials before current PIPAC protocols can be modified.^17^

By optimising intraperitoneal drug delivery, CapnoTip^®^ could help protect healthy peritoneal tissues from excessive exposure to cytotoxic agents, which is crucial for maintaining the peritoneum’s diverse protective functions.^18^ Minimising healthy tissue exposure to drug could help reduce local adverse effects, such as inflammation or fibrosis, and mitigate the risk of systemic side effects due to decreased drug absorption into the bloodstream.

The potential for lower chemotherapy dosing with CapnoTip^®^ aligns with a shift towards more targeted, patient-centred treatment that prioritises efficacy while minimising toxicity. Patients may experience improved tolerability, enabling them to complete more treatment cycles with fewer interruptions due to side effects. This approach could ultimately lead to better overall treatment outcomes, as patients may be more likely to adhere to the treatment schedule and tolerate therapy over longer durations.

In addition to improved drug delivery, CapnoTip^®^ achieved a significantly greater homogeneity within the peritoneal tissue, with no observable vertical gradient along the aerosolisation axis. This uniform distribution further supports the potential of CapnoTip^®^ to enhance therapeutic responses across challenging anatomical regions, such as the anterior abdominal wall and flanks.

Future studies will investigate the promising combination of CapnoTip^®^ with electrostatic PIPAC (ePIPAC) to further enhance therapeutic efficacy. Given CapnoTip^®^’s ability to achieve predominantly even drug spread, the distinct advantage previously attributed to ePIPAC in improving spatial drug uniformity^19^ may be reduced—or potentially rendered negligible—when used in conjunction with this advanced nebuliser. This raises important questions about the synergistic or complementary roles of both technologies, warranting further exploration in preclinical and clinical settings.

The study was conducted under controlled laboratory conditions simulating real-life operating room environments, employing clinically approved instrumentation and standardised protocols. To minimise ischaemic tissue alterations, bovine bladders were rapidly cooled and transported immediately postexplantation, followed by histological quality control to exclude necrotic samples. Pharmacological assessment was performed using the validated enhanced Inverted Bovine Urinary Bladder (eIBUB) model, which closely replicates the spatial and absorptive dynamics of the human peritoneal cavity. Previous investigations have established its suitability for preclinical PIPAC studies by demonstrating tissue penetration depths and local drug concentrations comparable to those seen in patients. While the eIBUB model lacks vascularisation—limiting its ability to assess systemic drug clearance and pharmacokinetics—this constraint applies equally to all test conditions within the model. Because both devices were evaluated under identical ex vivo settings, we consider the impact of absent systemic clearance to be consistent across comparisons, allowing for valid relative performance assessment.

In accordance with MDR 2017/745 requirements for usability and safety, no technical or safety concerns were identified during preclinical testing of the CapnoTip^®^ nebuliser. However, usability testing conducted as part of clinical validation revealed a limitation related to the hook-shaped impaction plate, which, in some cases, hindered the smooth removal of the device from the trocar. To address this, a protective sleeve was developed—a user-friendly tubular guide that fits over the impaction plate. This solution ensures safe, airtight insertion and removal, protects the trocar from damage, and enhances device stability and handling precision.

## Conclusions

The impaction-based PIPAC nebuliser CapnoTip^®^ significantly improved intraperitoneal drug delivery and distribution homogeneity compared to the CapnoPen^®^. These findings support the CapnoTip^®^ as a promising advancement in intraperitoneal chemotherapy, positioning it as a new benchmark in laparoscopic nebuliser technology for PIPAC, with strong potential to enhance therapeutic efficacy in treating peritoneal metastases. Consequently, the CapnoTip^®^, rather than the CapnoPen^®^, should be employed in future PIPAC clinical studies.

## Diclosure

This research was funded by CapnoPharm GmbH, Tübingen, Germany, and performed at the R&D Facility CapnoPharm GmbH, Tübingen, Germany. The patent DE102022104560A12023.08.31 protects CapnoTip^®^ and DE102017006185A1 – CapnoPen^®^. MAR, YS, and HS are employees of CapnoPharm GmbH, Tübingen, Germany. MAR has an equity interest in CapnoPharm GmbH.
